# The Circadian Rhythm of Breakthrough Pain Episodes in Terminally-ill Cancer Patients

**DOI:** 10.3390/cancers11010018

**Published:** 2018-12-24

**Authors:** Sara Campagna, Riccardo Sperlinga, Antonella Milo, Simona Sannuto, Fabio Acquafredda, Andrea Saini, Silvia Gonella, Alfredo Berruti, Giorgio Vittorio Scagliotti, Marco Tampellini

**Affiliations:** 1 Department of Public Health and Pediatrics, University of Torino, 10126 Torino, Italy; silvia.gonella@unito.it; 2 School of Nursing, Catholic University of the Sacred Heart, Cottolengo Hospital, 10152 Torino, Italy; riccardo.sperlinga@gmail.com (R.S.); ssannuto71@gmail.com (S.S.); fabioacquafredda84@gmail.com (F.A.); 3 FARO Foundation—Hospice Sergio Sugliano, 10121 Torino, Italy; antonella.milo@fondazionefaro.it; 4 Medical Oncology, University of Torino; Department of Oncology, San Luigi Gonzaga Hospital, 10043 Orbassano, Italy; a.saini@libero.it (A.S.); giorgio.scagliotti@unito.it (G.V.S.); marco.tampellini@unito.it (M.T.); 5 Department of Medical and Surgical Specialties, Radiological Sciences, and Public Health, Medical Oncology, University of Brescia, ASST-Spedali Civili, 25123 Brescia, Italy; alfredo.berruti@gmail.com

**Keywords:** analgesics, breakthrough pain, circadian rhythm, neoplasms, palliative care, quality of life

## Abstract

Opioid therapy must be adjusted to the rhythm of a cancer patient’s pain to ensure adequate symptom control at the end of life (EOL). However, to-date no study has explored the rhythm of breakthrough pain (BTP) episodes in terminally-ill cancer patients. This prospective longitudinal study was aimed at verifying the existence of a circadian rhythm of BTP episodes in terminally-ill cancer patients. Consecutive adult cancer patients at their EOL treated with long-acting major opioids to control background pain (Numeric Rating Scale ≤ 3/10) were recruited from two Italian palliative care services. Using a personal diary, patients recorded the frequency and onset of BTP episodes and the analgesic rescue therapy taken for each episode over a 7-day period. Rhythms identified in BTP episodes were validated by Cosinor analysis. Overall, 101 patients were enrolled; nine died during the study period. A total of 665 BTP episodes were recorded (average of 7.2 episodes, mean square error 0.8) per patient, with 80.6% of episodes recorded between 8:00 a.m. and 12:00 a.m. At Cosinor analysis, a circadian rhythm of BTP episodes was observed, with a Midline Estimating Statistics of the Rhythm (MESOR) of 1.5, a double amplitude of 1.8, and an acrophase at 12:30 p.m. (*p* < 0.001). Oral morphine was the most frequent analgesic rescue therapy employed. In terminally-ill cancer patients, BTP episodes follow a circadian rhythm; thus, tailoring the timing of opioid administration to this rhythm may prevent such episodes. This circadian rhythm of BTP episodes in terminally-ill cancer patients should be confirmed in larger samples.

## 1. Introduction

Breakthrough pain (BTP) is defined as “transitory exacerbations of pain that occur on a background of stable pain otherwise adequately controlled by around-the-clock opioid therapy” [1] or as “a transient exacerbation of pain that occurs either spontaneously, or in relation to a specific predictable or unpredictable trigger, despite relatively stable and adequately controlled background pain” [2]. BTP is generally categorized as “spontaneous” when unpredictable, and as “incident” when it is associated with an identifiable cause [3]. Although the clinical characteristics of BTP may change significantly among patients, typically BTP has a rapid onset (median interval to peak pain of 3 min), short duration (average of 30 to 60 min), high frequency (median: four episodes per day, range: one to >10 episodes per day), and a high severity [4,5].

In cancer patients, the prevalence of BTP ranges between 40% and 80% depending on the definition adopted and where the data were collected, with the higher prevalence reported in hospice [6]. A recent review of the most relevant national and international guidelines identified some differences in terms of the definition, diagnostic criteria, and treatment of BTP: generic cancer pain guidelines continue to support the use of oral opioids as an analgesic rescue therapy, while specific BTP guidelines promote transmucosal opioids for this purpose [7]. Cancer patients experiencing BTP have significantly greater pain-related functional impairment and psychological distress, [5] and a significantly reduced quality of life (QoL) compared to asymptomatic patients [8]. Furthermore, BTP highly impacts healthcare system expenditure, leading to more frequent medical visits and hospital admissions, and longer hospitalizations [9]. 

Several studies have investigated the relationship between pain and patients’ circadian timing (i.e., the rest-activity rhythm) [10,11,12]. Cancer symptoms can alter patients’ rest-activity rhythm. In turn, a disrupted rest-activity rhythm may increase pain perception in cancer patients [10,13], but patients with advanced lung cancer experienced improved rest-activity rhythms when their symptoms were addressed [11]. 

Neurobiologic investigations have demonstrated that body position can influence cortical response to pain somatosensory stimulation, with an overall decrease in pain sensitivity and an altered pain network outside the primary somatosensory cortex during head-down bed rest [14]. A horizontal resting position was shown to inhibit the fronto-parietal pain network, particularly at the central prefrontal regions typically involved in cognitive, affective, and motor aspects of pain processing [15]. Moreover, head-down bed rest can alter the diurnal rhythms of a number of physiological processes, some of which might be attributed to a reduction in autonomic nervous system activity [16]. 

Patients with advanced-stage cancer usually have a higher tumor burden and more frequent cancer-related symptoms, and they spend the majority of the day bedridden [17]. All these conditions increase a patient’s likelihood of experiencing alterations in their rest-activity rhythm, as well as in the occurrence of BTP episodes. The literature has shown a circadian rhythm of BTP episodes in cancer outpatients with a good performance status (PS) [18]. However, to-date no study has explored the rhythm of BTP episodes in terminally-ill cancer patients. Opioid therapy must be adjusted to the rhythm of a cancer patient’s pain to ensure adequate symptom control at the end of life (EOL). Symptom management is indeed one of the top-ranked quality indicators for hospice and palliative care identified by the American Academy of Hospice and Palliative Medicine and the Hospice and Palliative Nurses Association [19]. Therefore, this study aimed to verify the existence of a circadian rhythm of BTP episodes in terminally-ill cancer patients. 

## 2. Material and Methods

### 2.1. Study Design and Sample

In this multicenter, longitudinal, observational study, we recruited consecutive adult cancer patients at their EOL from two Italian palliative care services (one palliative home care service and one hospice) in the Northwest of Italy from December 2012 to July 2013. In order to be included, patients had to have (a) histologically confirmed diagnosis of cancer; (b) no possibility of active oncological treatment; (c) a life expectancy of less than 120 days; (d) analgesic treatment with a long-acting major opioids (i.e., fentanyl transdermal, morphine intravenous/intravenous elastomer/ subcutaneous/oral, oxycodone, methadone, hydromorphone, or buprenorphine); (e) their opioid dose assessed by previous titration; and (f) adequately controlled background pain (≤ 3/10 on the Numeric Rating Scale, NRS). We excluded patients receiving concomitant local analgesic treatment (i.e., peripheral nerve ablation or spinal cord treatment) at the time of recruitment, those who had competed radiation therapy or radionuclide therapy within one month of recruitment; those with an ascertained possibility of end-of-dose-related pain; those with major psychiatric disorders; and those with cognitive incompetence.

### 2.2. Procedures and Instruments

In each care setting, a palliative care nurse approached eligible patients and screened them according to the above-mentioned criteria. Screening took place during the daily visit for patients receiving home palliative care services and during hospice stay for hospice patients. All eligible patients were informed about the study and its aims, and they provided written informed consent. The study was approved by the Ethical Committee on 14 February 2013 (ethic code 3119/II/02/01).

Participating patients were asked to self-report the frequency and onset (day and time) of their BTP episodes and the analgesic rescue therapy (drug and route of administration) they took for each episode over a 7 day-period using a personal diary. Nurses and family caregivers helped patients with the reporting and checked that each BTP episode and analgesic rescue therapy was reported. BTP characteristics were assessed with a short form of the Italian version of the Alberta BTP [20]. 

At recruitment, we collected data on sex, age, and the following clinical characteristics: cancer site, metastasis (no vs. yes and site), Eastern Cooperative Oncology Group (ECOG) PS, around-the-clock analgesic therapy (drug and route of administration), QoL (Palliative Outcome Scale, POS), and care setting (home care vs. hospice). The ECOG identifies 5 grades of PS: 0 = no symptoms; 1 = mild symptoms; 2 = bedridden for ≤50% of waking hours; 3 = bedridden for >50% of waking hours; 4 = completely disabled; 5 = dead). The POS assesses a patient’s QoL by evaluating physical, psychological, and spiritual domains via 10 items. Eight items utilize a 5-point Likert scale, and the remaining two utilize a 3-point scale. The score for each item ranges from 0 (no problem) to 4 (severe problem). The overall POS score is obtained by summing the scores of all 10 items, and thus ranges from 0 (worst QoL) to 40 (best QoL) [21]. 

### 2.3. Statistical Analysis 

The categorical variables were computed as sums and percentages. Continuous variable were expressed as means and standard deviations (SDs). The chi-square test with Yates’ correction when needed, Mann-Whitney *U*-test, or *t*-test was used according to the nature of the variable.

Rhythms in BTP episodes were validated by two-way analysis of variance and by Cosinor analysis [22]. In the Cosinor analysis, a predetermined period (*τ* = 24h) is fitted to the cosine curves (*y*i = M + A cos (ϕ + θτi) + ei), and their significance is evaluated by the least-square method. This analysis yields three main parameters: Midline Estimating Statistics Of the Rhythm (MESOR) of the fitted cosine curve, defined as the average value of rhythmic function (e.g., cosine curve) fitted to data expressed in same units as the original data; acrophase (ϕ, occurrence of maxima in days or months, which may not take place at the time during which maximum concentration was observed); and double amplitude (the difference between the highest and lowest point of the fitted cosine curve). Correlation coefficient constant and mean square error (SEM) were used to determine the goodness-of-fit of the cosine curve. 

As the rhythm of pain may be influenced by patients’ health conditions [23], subgroup analyses were carried out by care setting (home care vs. hospice), site of metastases (bone vs. visceral only); and ECOG (≤2 vs. ≥3). Patients were also stratified by mean occurrence of BTP episodes into a lower BTP frequency group (≤7 BTP episodes per day) and higher BTP frequency group (>7 BTP episodes per day). Difference in QoL scores between the lower and higher BTP frequency groups was tested. Patients who died during the 7-day study period were excluded from the analyses.

All statistical computations were performed using the SPSS for Windows and STATISTICA for Windows software (StatSoft Inc., Tulsa, OK, USA). The level of statistical significance was set at 2-tail *α* level ≤ 0.05. 

## 3. Results

### 3.1. Population Characteristics

A total of 101 patients were enrolled. Nine patients died during the study period (one at Day 1, two at Day 3, one at Day 4, and five at Day 6). Therefore, 92 patients were included in the analyses: 44 in home care and 48 in hospice. Most patients were female (55.4%), mean age was 72.2 years, and PS in the study sample was poor (78.2% had ECOG ≥ 3). Lung cancer was the most common among our participants (17.5%), followed by cancer of the large bowel (15.2%). Thirty (32.6%) patients had metastasis of the bone, followed by metastasis of the liver (*n* = 20, 21.7%) and lung (*n* = 18, 19.6%) (Table 1). At the start of the study, the mean pain score was 1.9/10 (SD 1.2) on the NRS. Transdermal fentanyl was the most frequent long-acting opioid employed (*n* = 34, 37%), followed by intravenous morphine (*n* = 23, 25%), and oral oxycodone (*n* = 14, 15.3%) (Table 1). At baseline, the mean oral morphine equivalent dose administered to the patients was 132.6 mg. 

### 3.2. Breakthrough Pain Episodes and Analgesic Rescue Therapy 

A total of 665 BTP episodes were recorded. During the 7-day study period, only one patient reported no BTP episodes. Thirty-nine (42.4%) patients reported a total of 1 to 5 episodes, 33 (35.9%) reported 6 to 10 episodes, and 19 (20.7%) reported more than 11 episodes. Some patients reported experiencing up to five BTP episodes in the same day (Table 2). 

**Figure 1:**
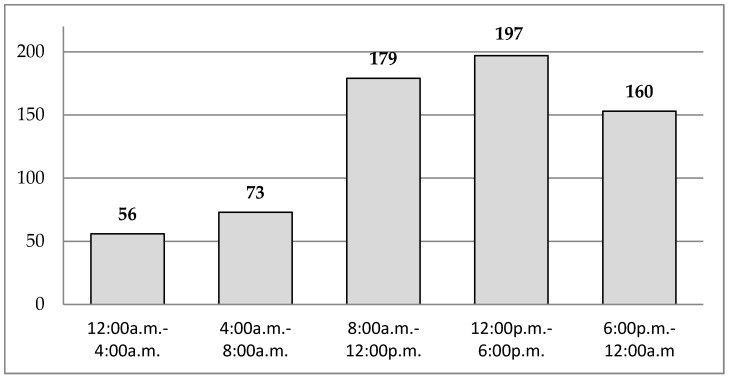
Total number of self-reported breakthrough pain episodes stratified by time frame.

The mean occurrence of BTP episodes across the study period was 7.2 (SEM 0.8) for each patient, with 80.6% of BTP episodes recorded between 8:00 a.m. and 12:00 a.m. (Figure 1). 

Oral morphine was the most frequent analgesic rescue therapy reported over the 7-day period, with 142 administrations; followed by intravenous morphine (88 administrations) and subcutaneous morphine (51 administrations). The daily number of analgesic rescue therapies employed ranged from 44 to 67 administrations across the 7-day period. Fourteen episodes were not treated (Table 3)

### 3.3. Rhythm Analysis 

**Figure 2:**
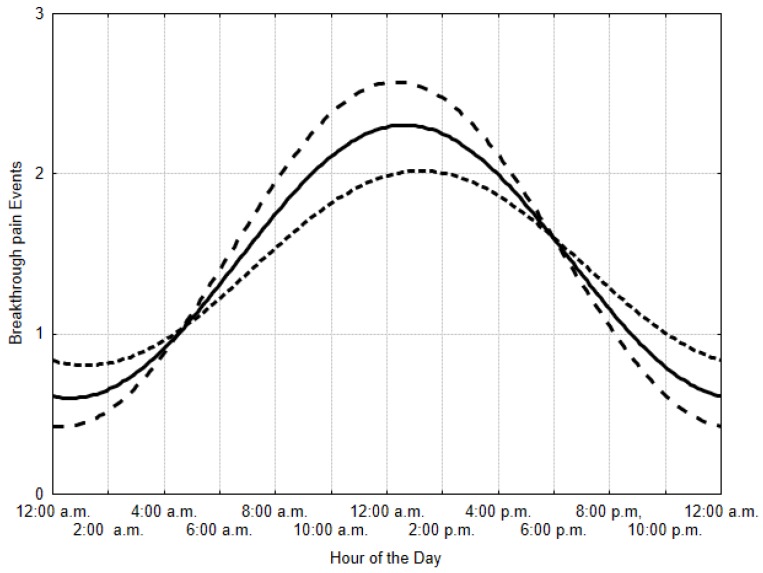
Pattern of breakthrough pain episodes. Note. All patients, solid line (MESOR 1.5; amplitude 0.9; acrophase at 12:30 p.m.; *p* < 0.001); patients with bone metastases (dashed line; MESOR 1.5; amplitude 1.1; acrophase at 12:15 p.m.; *p* < 0.001); patients with ECOG ≥ 3 (dotted line; MESOR 1.4; amplitude 0.6; acrophase at 1:00 p.m.; *p* < 0.01). Abbreviations: MESOR, Midline Estimating Statistics of the Rhythm; ECOG, Eastern Cooperative Oncology Group.

At Cosinor analysis, there was a significant circadian pattern of BTP episodes, with a MESOR of 1.5, a double amplitude of 1.8, and an acrophase at 12:30 p.m. (*p* < 0.001) (Figure 2). 

A significant circadian rhythm of BTP was also found in the subgroup analyses (all *p* < 0.01). BTP episodes showed an acrophase at 12:00 p.m. and 1:15 p.m. among patients in home care and hospice, respectively. BTP episodes showed an acrophase at 12:15 p.m. and 12:30 p.m. in patients with bone metastases and visceral metastases only, respectively. BTP episodes showed an acrophase at 12:15 p.m. in patients with an ECOG ≤ 2, and an acrophase at 1:00 p.m. in patients with an ECOG ≥ 3 (Figure 2). The circadian rhythm of BTP episodes persisted after stratifying by care setting (home care vs. hospice), site of metastases (bone vs. visceral only), and ECOG PS (≤2 vs. ≥3). 

### 3.4. Relationship between Breakthrough Pain Episodes and Quality of Life

**Figure 3:**
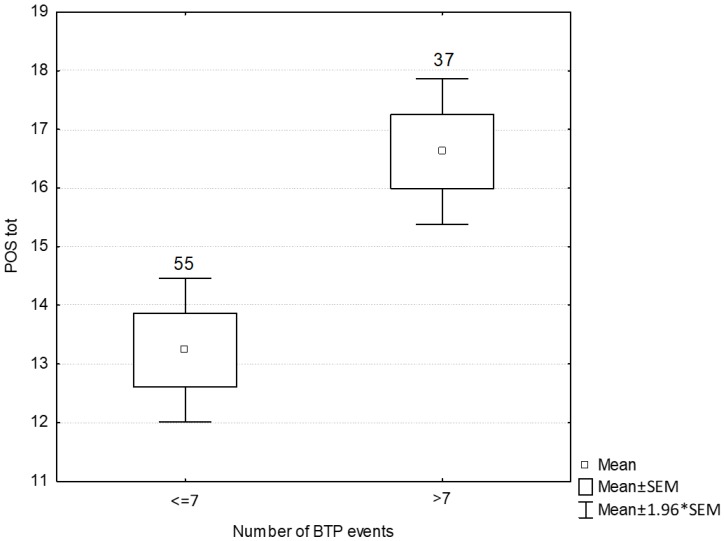
Quality of life scores according to the total number of breakthrough pain episodes (*p* < 0.001); Note. The number of patients belonging to each subgroup is reported on the top of the respective column; Abbreviations. BTP, breakthrough pain; SEM, mean square error, POS, Palliative Outcome Scale.

Fifty-five (59.8%) patients were in the lower BTP frequency group, and 37 (40.2%) were placed in the higher BTP frequency group. The higher BTP frequency group had higher POS scores than the lower BPT frequency group (mean ± SEM, 16.6 ± 0.6 vs. 13.2 ± 0.6, respectively, *p* < 0.001) (Figure 3).

## 4. Discussion 

To our knowledge, this is the first study to explore the circadian rhythm of BTP in terminally-ill cancer patients. The only existing study of the pattern of BTP in cancer patients was performed in outpatients with a good PS [18]. Our findings suggest that BTP episodes also follow a circadian rhythm in terminally-ill cancer patients, with a peak occurring in the late morning. This circadian rhythm was maintained in subgroup analyses, suggesting a common pathophysiology of pain patterns regardless of care setting, site of metastasis, or PS, with a comparable acrophase occurring between 12:00 p.m. and 1:00 p.m. This pattern indirectly confirms the results of previous studies, which reported an increased need for rescue analgesics in the daytime [24,25]. Therefore, health care professionals working in palliative care should establish the circadian rhythm of BTP episodes for each patient (for example by identifying the first BTP episode of the day) to tailor the timing of opioid administration, and thus potentially prevent the onset of BTP episodes. As the peak of BTP occurs in an established time of the day, it could be feasible to administer a supplementary fixed dose of immediate release morphine, calculated on the basis of the morphine equivalent of the around-the-clock amount of opioid, before the time of the peak. This intervention could help to significantly reduce patient’s BTP burden that in turn would be likely to translate in an improved QoL. Moreover, this intervention would not induce a worsening of opioid-related side effects since the supplementary dose consists of a conventional rescue dose for BTP without increasing the overall around-the-clock opioid dose. 

A previous work [18] hypothesized three mechanisms to explain the genesis of a circadian rhythm of BTP episodes in outpatients with cancer: (1) the presence of a circadian rhythm in physical activity; (2) a circadian rhythm in pain sensitivity; and (3) a circadian rhythm in drug pharmacokinetics or pharmacodynamics. However, although changes in patient position done by caretakers to administer care may partially account for predictable BTP occurrence in the morning [26,27], over three-quarters of our patients spent more than 50% of their time in a chair or bed (ECOG PS ≥ 3), making it challenging to explain their BPT episodes by a circadian rhythm in their physical activity. Moreover, sleep disruption is frequent in patients with advanced-stage cancer, especially those at their EOL, and night awakening increases BTP episodes [28]. Finally, palliative patients as ours usually have a very poor food intake due to cancer-induced hyporexia and tumour cachexia. Some of these patients receive intravenous or subcutaneous parenteral nutrition that do not outstretch gastroenteric organs [29,30]. Therefore, we believe that the impact of eating was very slight and should not have biased the results [31]. Consequently, it might be more plausible that the rhythm of BTP episodes is due to a circadian rhythm in pain perception and the pharmacodynamics of the analgesic drug rather than to a rhythm in patients’ physical activity or eating patterns. However, since patients with advanced-stage cancer lose the circadian rhythms of internal synchronizers such as cortisol and melatonin, it is likely that their whole temporal biological structure is altered. In addition, at recruitment, our patients received a high mean oral morphine equivalent dose (132.6 mg), which may conceal rhythms in BTP episodes.

Another, more intriguing explanation may be that put forth in the Zeitgebers theory [32]. According to this theory, all living systems have a “physiological clock” that allows them to maintain endogenous regulation through external cues known as Zeitgebers [32]. Zeitgebers can be physical stimuli (e.g., light), environmental stimuli (e.g., temperature), or social events, such as the timing of general activities or meals [33,34,35]. Recent progresses in neuroscience research have shown that our master circadian clock, which is located in the suprachiasmatic nucleus of the hypothalamus, controls day-dependent changes, such as the adrenal secretion of glucocorticoids, and coordinates various biological processes in peripheral tissues in a diurnal cycle-dependent manner [36]. Moreover, treatment strategies targeting the regulation of biological rhythms, such as sleep/wake cycles, eating patterns, activities, and social rhythms, were found to improve QoL [37]. Therefore, it seems reasonable to hypothesize that strong Zeitgebers maintain the circadian clock, even in terminally-ill patients, and this clock, in turn, maintains a circadian pattern in the rhythm of BTP. 

All our patients except one experienced at least one BTP episode: a close clinical examination that indicated adequately controlled baseline pain before the diagnosis of BTP was our cornerstone for defining pain [2]. This prevented us from misclassifying patients with uncontrolled background pain experiencing transient exacerbations of that pain from patients with controlled background pain experiencing episodes of BTP. Our patients had an average background pain intensity at recruitment of 1.9/10 on the NRS (SD 1.2), suggesting a good control of baseline pain. Our criterion to include only patients with a background pain intensity of ≤ 3/10 on the NRS was even more cogent than that of previous authors who enrolled patients with a basal pain of ≤ 4/10 on the NRS [38]. However, our prevalence of BTP was higher than that identified by a pooled analysis [6], likely because our participants were at the very EOL; almost 10% of our participants died during the 7-day observation period. With regard to the frequency of BTP episodes, a mean of four episodes per day is commonly considered acceptable [4]. Our patients reported a fewer BTP episodes per day compared to previous studies [38,39], with only a few patients reporting five episodes per day. This suggests that background analgesia was quite satisfactory and that better background analgesia may decrease the number of BTP episodes [40]. 

BTP episodes were most frequently treated using oral morphine (142 administrations), although BTP guidelines endorse the use of transmucosal opioids as rescue medications, and there is agreement in the preference of transmucosal fentanyl preparations to oral morphine when more rapid relief onset is necessary [1,41,42,43]. When ranking rescue medications by frequency of administration, sublingual fentanyl scored only fourth with 22 administrations across the 7-day period. 

Finally, our findings confirmed that BTP negatively impacts QoL, with patients reporting a higher frequency of BTP episodes having worse QoL scores. BTP affects QoL by increasing both physical and psychological distress: BTP interfered with patients’ daily activities by limiting their walking ability and negatively affected sleep and mood [39,44]. These data suggest that preventing BTP could improve patients’ well-being at EOL.

This study was conducted on a small sample, and future researchers should confirm the existence of a circadian rhythm of BTP episodes in larger samples of cancer patients at their EOL. Moreover, BTP episodes were self-reported; therefore some episodes may have been missed or incorrectly recorded in the diaries, particularly for those patients in palliative care at home. However, all patients had a family caregiver that could help them with the reporting, and a palliative care nurse checked the diary entries daily during routine visits. A strength of the study is the data collection on two different series of patients (patients in a palliative home care service and hospice patients), which reduced the possibility of a result by chance. Moreover, the circadian rhythm was analyzed using a specific, dedicated test. 

## 5. Conclusions

This study showed that, in terminally-ill cancer patients, BTP onset follows a circadian rhythm with a late-morning acrophase. These data provide a rationale for the prevention of BTP episodes, by identifying the pattern of BTP occurrence for each patient in order to tailor the timing of opioid administration. Future prospective randomized clinical studies should test whether the administration of short-acting opioids according to the specific patient’s BTP pattern can enhance around-the-clock analgesia. 

## Figures and Tables

**Table 1:** Patient characteristics at recruitment (*n* = 92).

Patient Characteristics	*n* (%)
**Male Sex**	41 (44.6)
**Age, Years**	
Mean (SD)	72.2 (12.0)
Range	41–97
**ECOG**	
1	13 (14.1)
2	7 (7.7)
3	28 (30.4)
4	44 (47.8)
**Site of the Primary Tumor**	
Lung	16 (17.5)
Large bowel	14 (15.2)
Breast	12 (13.0)
Urogenital	11 (12.0)
Gynecological	7 (7.6)
Head & Neck	7 (7.6)
Pancreas	6 (6.5)
Stomach	6 (6.5)
Other	13 (14.1)
**Site of Metastasis ^a^**	
Bone	30 (32.6)
Liver	20 (21.7)
Lung	18 (19.6)
Lymph nodes	16 (17.4)
Brain	6 (6.5)
Other	19 (20.6)
**Around-the-Clock Analgesic Therapy (Route of Administration)**	
Fentanyl (transdermal)	34 (37.0)
Morphine (intravenous)	23 (25.0)
Oxycodone (oral)	14 (15.3)
Morphine (intravenous elastomer)	8 (8.7)
Morphine (oral)	4 (4.3)
Methadone (oral)	4 (4.3)
Others ^b^	5 (5.4)

^a^ The sum is higher than the number several patients had more than one metastasis; ^b^ Buprenorphine transdermal (*n* = 2); Morphine subcutaneous (*n* = 2); Hydromorphone oral (*n* = 1); Abbreviations: SD, Standard deviation, ECOG, Eastern Cooperative Oncology Group.

**Table 2:** Episodes of breakthrough pain episodes over the 7-day period (*n* = 665).

Day		No. of Episodes	1	2	3	4	5	Overall No. of BTP Episodes/Day
Number of patients with BTP episodes/day (*N*)	Day 1	23	46	16	5	2	-	101
	Day 2	28	34	19	6	4	1	111
	Day 3	37	33	14	3	3	2	92
	Day 4	33	31	17	5	6	-	104
	Day 5	41	29	14	5	2	1	85
	Day 6	39	30	14	4	3	2	92
	Day 7	44	28	11	7	1	1	80

Abbreviations: BTP, breakthrough pain.

**Table 3:** Analgesic rescue therapy over the 7-day period.

Analgesics		Day 1	Day 2	Day 3	Day 4	Day 5	Day 6	Day 7	Overall Number of Administrations for Each Analgesic
Number of patients with analgesic rescue therapy/day (*N*)	Morphine (oral)	26	25	18	22	15	21	15	142
	Morphine (i.v.)	17	17	12	10	9	11	12	88
	Morphine (s.c.)	7	5	8	11	8	8	4	51
	Fentanyl (s.l.)	7	5	5	7	7	7	4	42
	Fentanyl (nasal spray)	3	4	4	2	2	3	4	22
	Methadone (i.v.)	2	2	1	3	4	2	3	17
	Paracetamol (i.v.)	1	2	3	3	1	-	-	10
	Morphine (s.l.)	2	-	1	-	1	-	1	5
	Ibuprofen (oral)	1	-	-	-	1	-	1	3
	Paracetamol (oral)	-	1	1	-	-	1	-	3
	Oxycodone (oral)	1	-	-	-	-	-	-	1
	Morphine (nasal spray)	-	1	-	-	-	-	-	1
	Ketorolac (oral)	-	-	1	-	-	-	-	1
	Ketoprofen (i.v.)	-	-	-	-	1	-	-	1
	Hydrocodeine (oral)	-	-	-	-	1	-	-	1
Overall number of analgesic administrations		67	62	54	58	50	53	44	388
Untreated episodes (*N*)		2	2	1	1	1	3	4	

Abbreviations: s.c., subcutaneous; s.l., sublingual, i.v., intravenous.
